# Developing a Youth-Led Digital Hypertension Education Intervention for Adults With Hypertension: Qualitative Study on Refinement and Acceptability

**DOI:** 10.2196/54909

**Published:** 2024-09-06

**Authors:** Sara W Heinert, Kelvin Guzman-Baez, Affan Aamir, Ananya Penugonda, Benjamin F Crabtree, Kathryn Greene, Carolyn J Heckman, Phillip Levy, Pamela Ohman Strickland, Shawna V Hudson

**Affiliations:** 1 Department of Emergency Medicine Rutgers Robert Wood Johnson Medical School New Brunswick, NJ United States; 2 Rutgers University New Brunswick, NJ United States; 3 Rutgers Robert Wood Johnson Medical School New Brunswick, NJ United States; 4 Department of Family Medicine and Community Health Rutgers Robert Wood Johnson Medical School New Brunswick, NJ United States; 5 Department of Communication Rutgers University New Brunswick, NJ United States; 6 Rutgers Cancer Institute Rutgers University New Brunswick, NJ United States; 7 Department of Emergency Medicine Wayne State University Detroit, MI United States; 8 Department of Biostatistics and Epidemiology Rutgers School of Public Health Piscataway, NJ United States

**Keywords:** hypertension, adolescents, adults, emergency department, digital health intervention, dyad intervention, intervention development, qualitative research, youth, adolescent, teen, teens, teenager, teenagers, adult, youth-led, digital health, health education, refinement, acceptability, USA, United States, care navigation, effectiveness, formative study, prototype, self-guided, online module, online modules, engagement, blood pressure, health knowledge, health promotion, nutrition education, support intervention, support, supports

## Abstract

**Background:**

Hypertension affects one-third of adults in the United States and is the leading risk factor for death. Underserved populations are seen disproportionately in the emergency department (ED) and tend to have worse blood pressure (BP) control. For adults, a lack of hypertension knowledge is a common barrier to hypertension control, while social support is a strong facilitator, and providing information that is culturally sensitive and relevant is especially important in this context. The youth experience increased confidence when given the responsibility to provide health education and care navigation to others. As such, we planned a randomized controlled trial (RCT) for the effectiveness of a digital youth-led hypertension education intervention for adult patients in the ED with hypertension, focusing on change in BP and hypertension knowledge.

**Objective:**

In preparation for an RCT, we conducted a formative study to determine acceptable and easily comprehensible ways to present hypertension information to adults with hypertension and optimal ways to engage youth to support adults on how to achieve better hypertension control.

**Methods:**

After creating an intervention prototype with 6 weekly self-guided hypertension online modules, we recruited 12 youth (adolescents, aged 15-18 years) for 3 focus groups and 10 adult ED patients with hypertension for individual online interviews to garner feedback on the prototype. After completing a brief questionnaire, participants were asked about experiences with hypertension, preferences for a hypertension education intervention, and acceptability, feasibility, obstacles, and solutions for intervention implementation with youth and adults. The moderator described and showed participants the prototyped intervention process and materials and asked for feedback. Questionnaire data were descriptively summarized, and qualitative data were analyzed using the template organizing style of analysis by 3 study team members.

**Results:**

Participants showed great interest in the intervention prototype, thought their peers would find it acceptable, and appreciated its involvement of youth. Youth with family members with hypertension reported that their family members need more support for their hypertension. Youth suggested adding more nutrition education activities to the intervention, such as a sodium tracker and examples of high-sodium foods. Adults discussed the need for a hypertension support intervention for themselves and the expected benefits to youth. They mentioned the overwhelming amount of hypertension information available and appreciated the intervention’s concise content presentation. They suggested adding more mental health and smoking cessation resources, information about specific hypertension medications, and adding active links for health care information.

**Conclusions:**

Based on focus groups and interviews with participants, a youth-led digital hypertension intervention is an acceptable strategy to engage both adults with hypertension and youth. Incorporating participant suggestions into the intervention may improve its clarity, engagement, and impact when used in a subsequent RCT.

## Introduction

In the United States, 33% of adults have hypertension [1], and blood pressure (BP) control has recently declined [2]. Hypertension is a leading risk factor for death [3], stroke [4], renal dysfunction [5], and heart disease [6]. Significant racial and ethnic disparities in hypertension morbidity and mortality outcomes exist [7,8]. A lack of hypertension knowledge is a common barrier to hypertension control [9-11], while social support is a strong facilitator [9,11,12], and providing information that is culturally sensitive and relevant is especially important [11,13].

Youth have shown increased confidence when given the responsibility to provide health education and care navigation to others [14-16]. Guided by the Social Cognitive Theory [17], youth who contribute to health programs can expand their knowledge and increase their healthful decision-making capacity [16,18-20].

As such, we designed a youth-led hypertension digital education intervention for adult patients in the emergency department (ED) with hypertension, focusing on hypertension knowledge and change in BP. We targeted adults in the ED because underserved populations are seen disproportionately in that setting [21] and tend to have worse BP control [2]. We aimed to determine acceptable and easily understood ways for youth to present information to adults with hypertension.

## Methods

### Overview

We developed a youth-led hypertension electronic education tool to guide youth through learning and then teach adults how to control hypertension using material from reputable sources (eg, Centers for Disease Control and Prevention) that was publicly available in English and Spanish. The intervention prototype had 6 weekly self-guided online modules housed in REDCap (Research Electronic Data Capture [Vanderbilt University]) [22,23], each with a theme (eg, healthy eating). Textbox 1 shows the first module prototype.

**Example module for digital youth-led hypertension education intervention prototype.**

**Week 1: What is Hypertension?**

**Please complete the module below in the next 7 days.**

1. Select which language you would like to receive the material in (English, Spanish, or both) based on the language preference of your adult.
2. Next, go through the material yourself and answer the questions provided.
3. Then, get together with your adult with hypertension. Walk the adult through the material, paying attention to the information in the questions you answered.
4. Finally, work with your adult to complete the “Evidence to Submit” for the module and submit your work in the module. Some modules will have options where you can choose which to complete and some modules will only have one option.

**OBJECTIVES**

This week, learners will know…

1. The definition of high blood pressure.
2. What blood pressure numbers are considered “high”.
3. Health consequences from high blood pressure.
4. Ways to lower high blood pressure.

In which language would you like to view the materials? (English, Spanish, Both)

**STEP 1. Review the materials below.**

Please note that the video has old high blood pressure cutoff values. High blood pressure is now 130/80 and above.

- *“CDC Vital Signs: Getting Blood Pressure Under Control” video from the Centers for Disease Control and Prevention (CDC)* [24]
- *“Blood Pressure: Know Your Numbers” infographic from CardioSmart: American College of Cardiology* [25]

**STEP 2. Answer these questions on your own and use them to help you guide your adult through the material.**

1. What is blood pressure?
2. Above what blood pressure value is considered to be high blood pressure?
3. Name one negative health problem that high blood pressure can cause.
4. Name one lifestyle change that can help lower blood pressure.

**STEP 3. Evidence to Submit**

Fill in the worksheet below with your adult and submit the completed worksheet (in whatever format works best for you, such as pdf, screen shot, or taking a picture).

- “Lower Your Blood Pressure: Make the Most of Your Appointment with a Health Care Professional” worksheet from the American Heart Association and American Medical Association [26]

Youth (aged 15-18 years) were recruited for 3 focus groups with 12 total participants (3-5 per group) from summer programs in New Jersey and Chicago. We recruited 10 adults with hypertension from the Robert Wood Johnson University Hospital ED in New Brunswick, New Jersey. Focus groups were conducted from October 2021 to April 2022, and interviews from February to July 2022, both over Zoom (Zoom Video Communications). After completing a brief REDCap questionnaire, participants were asked about hypertension experiences, hypertension education intervention preferences, acceptability, feasibility, obstacles, and solutions for intervention implementation. Participants were shown the prototyped intervention materials and provided feedback. Focus groups lasted ~90 minutes, and interviews lasted 38-85 minutes and were audio recorded.

Questionnaire data were descriptively summarized. Audio recordings were transcribed verbatim and analyzed using a template organizing style for analysis [27] by 3 study team members using NVIVO (Lumivero).

### Ethical Considerations

The study was approved by the Rutgers University Institutional Review Board (Pro2021000126). Participants received an information letter about the study, adults signed informed consent forms and signed parental permission, and youth assent was required for 15- to 17-year-olds. Participants were assigned study ID numbers to deidentify their responses. Participants received US $20 gift cards.

## Results

### Participant Characteristics

Demographic data are provided in Table 1.

**Table 1:** Characteristics of participants in focus groups and interviews about acceptable ways for youth to present information to adults in the ED with hypertension (N=22), New Jersey and Chicago (Youth) & New Jersey (Adults), 2021-2022.

				Youth, n (%)		Adults with HTN^a^, n (%)
**Demographics**						
	**Sex**					
		Female	7 (58)		4 (40)	
		Male	5 (42)		6 (60)	
		Age (years), mean (SD), range	15.9 (0.8), 15-17		49.8 (17.0), 21-75	
	**Race**					
		Asian	1 (8)		4 (40)	
		Black	1 (8)		1 (10)	
		White	4 (33)		4 (40)	
		Other^b^	7 (58)		1 (10)	
	**Ethnicity**					
		Hispanic	9 (75)		2 (20)	
		Non-Hispanic	3 (25)		8 (80)	
	**Primary language**					
		English	11 (92)		9 (90)	
		Spanish	1 (8)		0 (0)	
		Other (Tagalog)	0 (0)		1 (10)	
	**Insurance type (n=9)**					
		Medicare	—^c^		1 (11)	
		Medicaid	—		2 (22)	
		Private insurance	—		6 (67)	
	**Highest level of education**					
		Some high school	—		1 (10)	
		High school degree	—		4 (40)	
		College degree	—		2 (20)	
		Graduate degree	—		2 (20)	
		Trade school	—		1 (10)	
**Medical history**						
	Years ago, doctor told participants they had high BP^d^ (HTN), mean (SD)			—		6.4 (4.1)
	Takes medication for high BP			—		8 (80)
	Has a primary care doctor and seen a doctor in the past year			—		9 (90)
**Technology**						
	Has access to a data plan or internet and a smartphone or computer			12 (100)		9 (90)
**Youth**						
	Has a youth living in their home who is 15-18 years old			—		1 (10)
**Family HTN history**						
	Has family member(s) with HTN (n=11)			8 (67)		6 (60)
	**Type of family member(s) with HTN (can choose multiple)**					
		Parent	4		6	
		Grandparent	7		2	
		Sibling	0		2	
		Aunt/Uncle/Cousin	3		1	
**Self-reported HTN knowledge, on a scale of 1 to 10, how much do you know about**						
	**High BP**					
		1-3 (least)	3 (25)		2 (20)	
		4-6 (moderate)	6 (50)		2 (20)	
		7-10 (most)	3 (25)		6 (60)	
	**What you can do to control high BP**					
		1-3 (least)	4 (33)		1 (10)	
		4-6 (moderate)	3 (25)		4 (40)	
		7-10 (most)	5 (42)		5 (50)	
**What information would you want to know about high BP that you do not know?^e^**						
	Causes/risk factors			5		0
	Long-lasting effects/consequences			4		0
	Symptoms			2		0
	Ways to control BP			2		4
	Medication to treat			2		0
	How it affects the body			2		1
	Other			1		1
**What are some barriers you or someone you know has for controlling high BP?^e^**						
	Barriers to eating healthy foods			4		2
	Forgetting to check BP/take meds			2		1
	Consistency/habits			0		2
	Lack of knowledge on how to control BP			2		1
	Cost/insurance (including medication)			0		3
	Unknown			2		0
	Other			3		4

^a^HTN: hypertension.

^b^For adults, “Other” includes Hispanic/Latino/Mexican American (n=3), unspecified (n=3), American Indian/Alaskan Native (n=1). For youth, “Other” includes more than one race (n=1).

^c^Not applicable.

^d^BP: blood pressure.

^e^These questions were open-ended and answers were coded. One participant’s answer may fit multiple categories.

### Hypertension Knowledge

Youth and adults wanted to learn tangible ways to control BP. Youth wanted to help adult family members with hypertension, and adults wanted information about hypertension risk factors and eating habits to improve BP.

…my dad and a lot of his family have high blood pressure…my dad passed with a stroke, because it wasn’t treated…I didn’t really know how to speak to him. But I feel like this intervention really helps.

Youth #7

I don’t know the root cause of why or how I got it. Or is there anything that I could have done to prevent it?...Now that I have it, I know that this is not reversible, but how best can I control that?

Adult #5

### Role of Doctor

Youth indicated that adults in their family prefer learning about hypertension from their doctor but witnessed problems, such as (1) adults not knowing how to talk to a doctor about hypertension, (2) doctors telling adults about healthy habits without explaining how to implement them, (3) communication challenges due to language differences. While many adults had positive interactions with their doctor, some challenges included (1) quick appointments, (2) a tendency to suggest medication before lifestyle changes to control BP, and (3) mentioning what to do to control BP but not how to implement recommendations.

…give them examples of what they can talk about, because sometimes they might not realize that that’s something that they could be talking to a doctor about...

Youth #2

…[my doctor] is very fast. She has too many patients.... and every time I tried asking a question to her... It feels like I am bothering her.

Adult #8

…the doctor was like, oh, keep your sodium below 2%…and I’m like I don’t even understand what that means…I had to go and learn how to read all these labels on my own, and try to figure out what to eliminate from there… you can’t always tell someone to go research it on their own, because they don’t understand how to find a reputable resource in terms that they understand...

Adult #4

### Role of Youth

Both adults and youth appreciated involving youth in the intervention and believed they are integral to increasing healthy behaviors for adults with hypertension. Youth recognized that adults trust them, making it easier for adults to ask them questions about hypertension. Adults also appreciated the hands-on learning to get teenagers thinking about hypertension.

…it’s like about their close family members. I think they’re definitely interested because it affects them, like how they live and everything about that. So, because it’s not like a random person they wouldn’t be interested in.

Youth #8

…getting the education early would be helpful…because the older you are, the more set in your ways you are…But having someone who’s able to support you and be able to provide you with other resources to educate yourself or even just support you through that journey would be helpful, like, “Here mom, I know you don’t like changing eating this and that’s because you like how it tastes. I found something else we can use to take some of that salt out and you can still enjoy it.”

Adult #4

### Positive Intervention Feedback

Youth and adults said the intervention was comprehensive, digestible, visually appealing, and inclusive, and they liked hypertension “tools” (eg, medication log). Youth liked the manageable steps that motivate healthy living. Adults said online hypertension information is overwhelming, so they appreciated having concise information from reputable sources. They liked that the intervention encourages patients to talk with their doctors and take more active roles in their health.

Involving the youth would help them…really keep track of their medication and make sure that they’re taking it in order to keep them healthy.

Youth #6

You do research online. There are tons of articles…many are written by people with their own sets of opinions, and you try to weed out and make sense of it… and then you start feeling more frustrated…you feel like you’re contributing to your disease more…I’m trying to figure this out by myself, but if it’s all available in one place in one package, it would be fantastic because I don’t have to A) search for it, and B) worry if it’s from a good source or not because sometimes it’s quite easy to get misinformation…

Adult #5

### Barriers to Completing Intervention

Youth were concerned about timing and competing priorities for their adult and said they could guide adults who are uncomfortable with technology. Adults stressed that everyone is different, so not all adults with hypertension will be motivated to participate.

You can have 20 people in the same room and everybody’s gonna have a different reaction to the same thing…

Adult #10

…the way you’re setting this up, it’s great, but you know you could lead a horse to water but gotta be able - on their own...

Adult #7

### Suggested Changes to Intervention Prototype

Although enthusiastic, participants suggested improvements to modules (Table 2).

**Table 2:** Suggestions by youth and adults with hypertension in focus groups and interviews to incorporate into a digital youth-led hypertension education intervention.

Module and suggestion			Group who suggested		Change made
**Overall**					
	Should add a progress bar so that the youth know how far they’ve gone with the modules and how many modules are remaining.	Adult		Information has been added to each module that includes X of 6 so that participants know how far they have to go.	
	Participants were interested in videos that explained the intervention process and the ability to have a discussion or ask questions about the material.	Youth		If needed, consider adding an FAQ^a^ document or links to videos in the intervention (live interactive elements would be difficult for this study but could be considered for future studies).	
	Should make questions easier to answer.	Adult		Changed open-ended questions to multiple choice.	
**Healthy eating**					
	Include a list of specific foods to avoid and incorporate into meals.	Youth		Added “The Salty Six” material to Healthy eating module.	
	Could add a sodium tracker to become aware of how much sodium consumed in a day.	Youth		Added a sodium tracker to the Healthy Eating module (as an option for submission of evidence).	
	Add something where you can track calories (and know how many calories are in foods).	Youth		Added to optional material: If you are interested in apps that help you track calories, there are several that are free and available in English and Spanish. Some examples are MyFitnessPal: Calorie Counter and MyPlate Calorie Counter.	
	“an additional resource about when you have to eat out, where you can gear yourself towards lower sodium choices”	Adult		Add info that includes FDA’s^b^ “Make lower-sodium choices at restaurants” as optional material (in English and Spanish).	
**Coping with stress and smoking**					
	The smoking module should offer resources to help people quit smoking.	Adult		Added additional smoking resources from the CDC^c^ about how to quit smoking.	
	Stress modules should include mental health resources for people having trouble reducing stress.	Adult		Added additional mental health resources from SAMHSA^d^.	
	“Quitting smoking is difficult and should be approached with sensitivity.”	Adult		Added a sensitivity warning to this module.	
**Taking my medicine**					
	The participant didn’t see the names of his BP^e^ meds on the medication module and was interested in learning about their meaning.	Adult		Added to optional material information from AHA^f^ which lists and provides information for each type of BP meds.	
**Talking to a doctor**					
	The participant would also like easy-to-access resources on health centers, their locations, and their hours rather than a PDF, which may not be dynamic enough of a resource because health center information can change.	Adult		Added active links to this module so that participants can get up-to-date info.	

^a^FAQ: frequently asked questions.

^b^FDA: Food and Drug Administration.

^c^CDC: Centers for Disease Control and Prevention.

^d^SAMHSA: Substance Abuse and Mental Health Services Administration.

^e^BP: blood pressure.

^f^AHA: American Heart Association.

## Discussion

### Principal Findings

Youth and adults showed great interest in the intervention prototype and thought that their peers would find it acceptable and appreciated youth involvement. The key recommendation to refine the intervention is that the youth said that their family members with hypertension need more support. The youth suggested adding a sodium tracker and examples of high-sodium foods. Adults mentioned the overwhelming amount of hypertension information available and appreciated the intervention’s conciseness and its support for doctor interactions. Adults suggested adding more mental health and smoking cessation resources, information about specific hypertension medications, and active links for health care information.

We could find no previous studies to develop a hypertension intervention for adults involving youth. However, studies have shown that youth can be a source of information and influence their parents’ health decision-making [28,29], mirroring the positive reactions of our participants.

### Limitations

Our sample size was modest, and English-speaking adults with hypertension were recruited from a single ED, which may limit generalizability. Additionally, youth participants were not required to know an adult with hypertension; however, having this eligibility criterion might have allowed for richer data from the youth.

### Conclusions

We refined the intervention to reflect participant input before use in a planned RCT of adult-youth dyads. Incorporating participant suggestions into the intervention will likely improve its clarity, engagement, and impact.

## Abbreviations

BP: blood pressure
ED: emergency department
RCT: randomized controlled trial

## Data Availability

The datasets generated or analyzed during this study are available from the corresponding author upon reasonable request.
